# Advancing our understanding of the influence of drug induced changes in the gut microbiome on bone health

**DOI:** 10.3389/fendo.2023.1229796

**Published:** 2023-10-09

**Authors:** Stacyann Bailey, Keith Fraser

**Affiliations:** ^1^ Department of Biomedical Engineering, University of Massachusetts Amherst, Amherst, MA, United States; ^2^ Institute for Applied Life Sciences, University of Massachusetts Amherst, Amherst, MA, United States; ^3^ Department of Biological Sciences, Rensselaer Polytechnic Institute, Troy, NY, United States; ^4^ Center for Biotechnology and Interdisciplinary Studies, Rensselaer Polytechnic Institute, Troy, NY, United States

**Keywords:** Osteoporosis management, microbiome and health, bone mineralization, pharmacomicrobiomics, microbiome-derived metabolites

## Abstract

The gut microbiome has been implicated in a multitude of human diseases, with emerging evidence linking its microbial diversity to osteoporosis. This review article will explore the molecular mechanisms underlying perturbations in the gut microbiome and their influence on osteoporosis incidence in individuals with chronic diseases. The relationship between gut microbiome diversity and bone density is primarily mediated by microbiome-derived metabolites and signaling molecules. Perturbations in the gut microbiome, induced by chronic diseases can alter bacterial diversity and metabolic profiles, leading to changes in gut permeability and systemic release of metabolites. This cascade of events impacts bone mineralization and consequently bone mineral density through immune cell activation. In addition, we will discuss how orally administered medications, including antimicrobial and non-antimicrobial drugs, can exacerbate or, in some cases, treat osteoporosis. Specifically, we will review the mechanisms by which non-antimicrobial drugs disrupt the gut microbiome’s diversity, physiology, and signaling, and how these events influence bone density and osteoporosis incidence. This review aims to provide a comprehensive understanding of the complex interplay between orally administered drugs, the gut microbiome, and osteoporosis, offering new insights into potential therapeutic strategies for preserving bone health.

## Introduction

Osteoporosis is a progressive systemic skeletal disorder characterized by reduced bone mass, weakened bone structure, and increased risk of fractures (1). This condition affects millions of people worldwide, with older adults and postmenopausal women being the most vulnerable population (2–4). Osteoporosis can be classified as a “silent disease” because it can progress without symptoms until a fracture occurs. The most common fractures associated with osteoporosis occur in the hip, spine, and wrist, which can lead to significant morbidity, reduced quality of life, and increased mortality.

**Figure 1:**
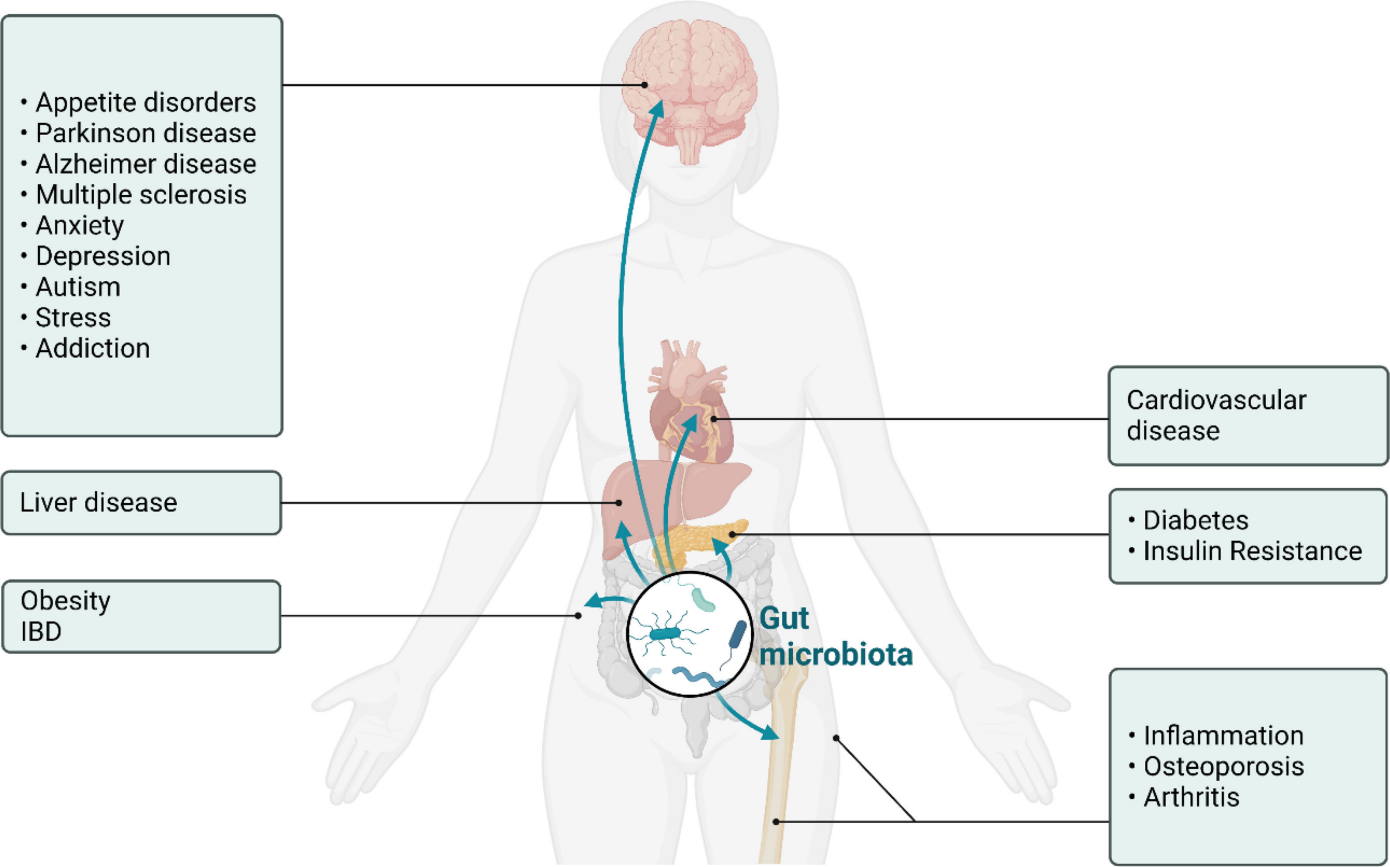
Schematic of the physiological impact of dysbiosis in the gut microbiome in humans. Shown here is the ability of molecules produced in the gut to influence neurodevelopmental, neurodegenerative, metabolic, cardiovascular, and inflammatory disease states.

Various factors contribute to the development and progression of osteoporosis, including genetic predisposition, hormonal imbalances, nutritional deficiencies, and lifestyle choices. Recently, the gut microbiome has emerged as a significant player in human health (5), with mounting evidence demonstrating how gut dysbiosis contributes to the development of disease that include obesity (6–11), diabetes (6, 8, 12–14), autoimmune disease, inflammatory bowel disease (IBD) (15–17), cancer (18–22), neurodegenerative diseases (23–26), neuropsychiatric disorders (27–31), cardiovascular disease (30, 32–35), and osteoporosis (36–41) (**Figure 1**). In each of these cases metabolites produced in the gut can induce physiological changes at distant anatomical locations via routes such as the gut-brain axis, the gut-bone axis and enterohepatic signaling (42–49). The gut microbiome refers to the diverse ecosystem of microorganisms, including bacteria, fungi, and viruses, residing in the gastrointestinal tract that communicate with host cells (50–55). This community of microorganisms is also highly dynamic, with the composition changing over time and in response to various factors such as diet (56, 57), pharmacological intervention (58–64), and other environmental factors (55, 65). Homeostasis in the gut microbiome is crucial for maintaining the balance between health and disease and can be achieved by regulating digestion, nutrient absorption, immune system modulation, and synthesis of essential vitamins and metabolites (52, 66–69).

Recent studies have begun to elucidate the mechanisms through which dysbiosis in the gut microbiome influences bone health. This relationship is primarily mediated by microbiome-derived metabolites and signaling molecules that affect bone mineralization (36, 37, 39). Moreover, immune cell activation induced by changes in cellular diversity and metabolism in the gut microbiome has also been shown to affect bone density. In this review we will explore how chronic disease and orally administered drugs stimulate the development and progression of osteoporosis via dysbiosis in the gut microbiome. The use of orally administered drugs, including antimicrobial and non-antimicrobial agents, can disrupt the gut microbiome’s diversity, physiology, and signaling (61, 63, 70), leading to changes in bone density and osteoporosis incidence. Understanding the complex interplay between orally administered drugs, the gut microbiome, and osteoporosis is crucial in developing effective therapeutic strategies for the prevention and treatment of this debilitating condition, particularly in high-risk populations.

Orally administered drugs, especially non-antimicrobials are regularly used to treat chronic diseases that themselves can perturb the gut microbiome and influence osteoporosis incidence. Consequently, we will explore how these medications can exacerbate or, in some cases, treat osteoporosis. Finally, we will propose potential therapeutic strategies targeting the gut microbiome to mitigate the effects of orally administered drugs on osteoporosis and outline future research directions in this area.

## Gut microbiome and bone health

**Figure 2:**
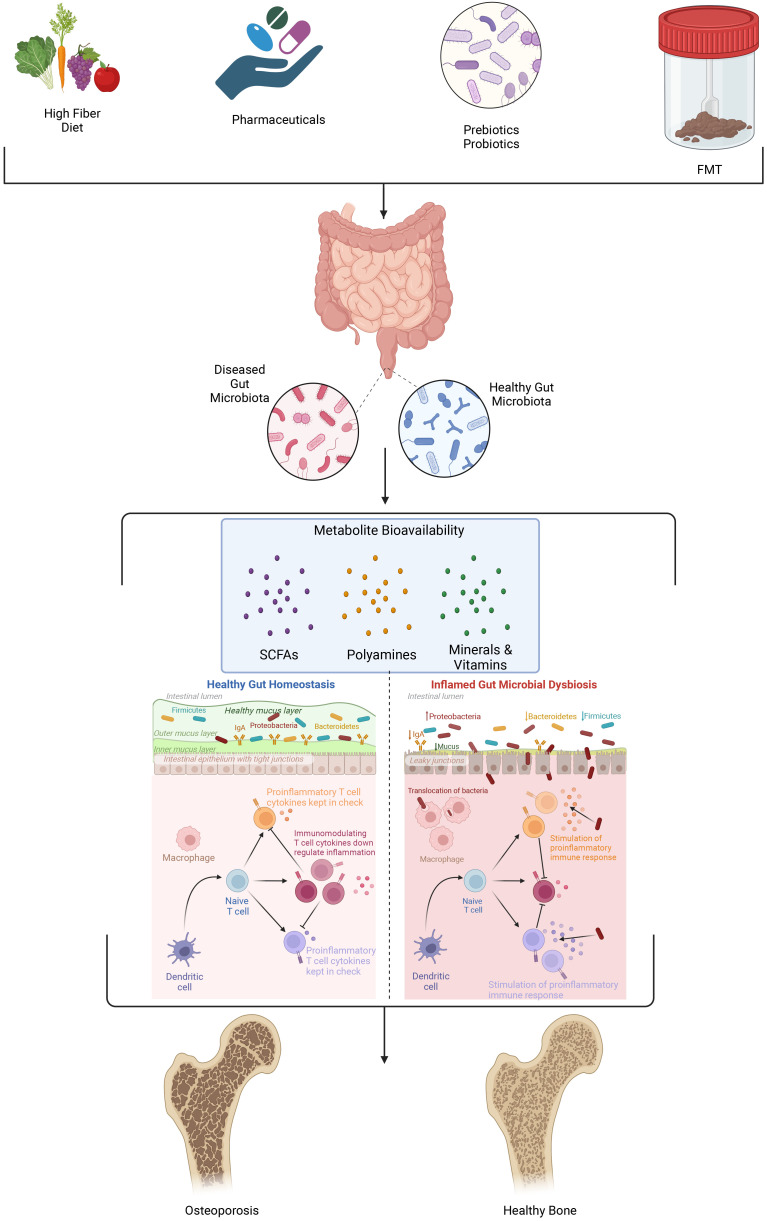
Several factors including diet, pharmaceutical use, pre- and probiotic consumption and fecal microbial transplant (FMT) influence the composition, diversity, and metabolic profile of microorganisms present in the gut microbiome. As the organismal mix and metabolic landscape in the gut microbiome shifts the permeability of the intestinal lining becomes compromised, gut metabolites and signaling molecules enter into systemic circulation and perturb immune signaling. Collectively these changes accelerate bone resorption that is the hallmark of osteoporosis.

The gut microbiome is a complex and diverse ecosystem consisting of trillions of microorganisms, including bacteria, fungi, and viruses (55, 71). This microbial community plays a crucial role in maintaining human health by participating in various physiological processes such as digestion, nutrient absorption, immune system regulation, and synthesis of essential vitamins and metabolites. Recent evidence suggests that the gut microbiome also has a significant impact on bone health, with alterations in microbial diversity and composition being associated with changes in bone density (72–74). A balanced gut microbiome contributes to optimal bone health by promoting the efficient absorption of essential nutrients, including calcium and phosphorus, which are critical for bone formation and remodeling (**Figure 2**). It also plays a role in the regulation of immune and metabolic homeostasis (75). Conversely, dysbiosis in the gut microbiome, characterized by reduced diversity and shifts in microbial composition, can lead to malabsorption of metabolites and vitamins (polyamines, SCFAs, calcium and vitamin D, inflammation, and subsequent bone loss (**Figure 2**). This relationship between gut microbiome diversity and bone density underscores the importance of maintaining a healthy and diverse microbial flora for optimal bone health.

Sjögren et al. (2012) demonstrated a link between the gut microbiome and bone health. Female C57BL/6J germ-free mice exhibited increased bone mass and fewer osteoclasts compared to conventionally raised (CONV-R) mice (76). These germ-free mice also had reduced osteoclast precursor levels, CD4+ T cells and inflammatory cytokines compared to CONV-R mice. In a follow-up study by Li et al. (2016) involving C57BL/6J germ free (GF) mice it was reported that the gut microbiome could mediate bone mass reduction during sex steroid deficiency-induced inflammation (77). While Li et al. observed no bone loss during sex steroid deficiency in GF mice they observed an in increase in the production of osteoclastogenic and inflammatory cytokines such as TNF-α, RANKL, and IL-17 during sex steroid deficiency in animals with healthy gut microbiomes. These data supported Sjogren’s findings that the gut microbiome was capable of amplifying bone resorption by elevating inflammatory and osteoclastogenic cytokine levels.

In experiments involving GF mouse models the expression of TNF-α and RANKL was reduced compared to CONV-R wildtype (WT) mice. This effect was lost in Nod1^-/-^ and Nod2^-/-^ mice when the GF animals were colonized with microbiota from the CONV-R WT mice. The observed shift in signaling indicated that microbiota induced increases in TNF-α and RANKL expression which modulated reductions in bone mass and is dependent on both Nod1 and Nod2 signaling (78). Additionally, bone marrow-derived mesenchymal stem cells (BMMCs) in GF mice displayed high proliferation and osteogenesis, but colonization with specific pathogen-free (SPF) mice microbiota normalized BMMCs proliferation and reduced osteogenesis (79).

Antibiotic studies further supported the gut microbiome’s role in inducing bone resorption, with low-dose antibiotics increasing bone mineral density (BMD) in mice. However, results are complex due to factors such as mouse strain and gender. Some studies on male mice of other strains suggested that the gut microbiome might induce bone resorption instead. Lactobacillus plantarum supplementation stimulated growth hormone (GH) activity in male BALB/c GF mice, promoting juvenile growth and preventing stunted growth during chronic undernutrition (80). In contrast, antibiotic use in male C57BL/6J mice reduced bone mineral content, unlike in female C57BL/6J mice (81). Yan et al. reported that long-term colonization of CB6F1 GF mice with SPF mice microbiota increased bone mass, whereas antibiotic treatment inhibited bone formation. Yan et al.’s study demonstrated that the gut microbiome’s net effect on bone depends not only on the strain but also on the duration of colonization. They found that short-term colonization (one month) significantly decreased bone mass, while long-term colonization (eight months) promoted bone formation. Colonization increased IGF-1 serum levels, which inhibited bone formation in the short term due to osteoclast promotion but induced bone formation during long-term colonization. Additionally, short-term colonization increased osteoclastogenic cytokines such as RANKL, TNF-α, and IL-1β in both the colon and bone marrow (Yan et al., (72)).

Quach et al. (82) reported that gut microbiome reconstitution did not affect bone health in GF mice, potentially due to differences in colonization methods, animal facilities, or sources of germ-free mice. Studies have shown that animals from different facilities carry distinct gut microbiomes, which might influence the colonization effect on bone health. A similar level of interpersonal diversity is observed in humans due in part to genetic and non-genetic factors such as demographics, diet, lifestyle, exposure to xenobiotics, and pollutants on gut microbial diversity (83–87). Collectively, these studies suggest that the gut microbiome can have both catabolic and anabolic effects, depending on the strain, gender, and colonization duration. These findings may also apply to humans, as factors such as birth method, genetics, sex, diet, pharmaceuticals, and other environmental influences can affect gut microbiome composition, a known modulator of bone health.

Various studies have since further demonstrated the gut microbiome composition’s impact on bone health. Undernourished children, for example, exhibit altered gut microbiome composition. Blanton et al. (88) found that transplanting microbiota from undernourished children or infants to germ-free mice induced growth impairments in the recipient mice. Conversely, cohousing GF mice that received microbiota from undernourished children with those receiving microbiota from healthy children prevented growth impairments (88). This evidence supports the idea that microbial diversity determines the gut microbiome’s overall effect on bone health. Other studies have shown that changes in gut microbiome composition can impair bone mechanical properties and affect bone strength (41). Rios-Arce et al. (89) reported that antibiotic-induced dysbiosis caused bone loss in mice, while *Lactobacillus reuteri* administration restored gut microbiome composition and alleviated bone loss. A recent study by Ma et al. (90) highlighted that transplanting gut microbiomes from young rats to aged rats with senile osteoporosis improved bone loss by restoring the aged rats’ gut microbiome composition. This restorative phenotype was brough about by adjusting the ratio of Firmicutes/Bacteroidetes and higher abundance of *Helicobacter* and *Prevotella*. Collectively, these studies have served to advance the field of osteomicrobiology as the mechanisms through which the gut microbiome influences bone health continue to be identified.

## Gut immune signaling and bone health

Recently, studies have shown that gut microbiome-derived compounds such as Urolithin-A can activate autophagy in bone marrow macrophage resulting in decreased osteoclastic bone resorption in a mouse model of senile osteoporosis (91). The flavanol kaemporal has also been shown to promote proliferation, differentiation, and mineralization of osteoblasts but in higher concentrations (>50 μM) can induce autophagy that induces a decline in bone mineralization (92). The gut microbiome can influence bone health by modulating local and systemic immune responses (76, 77). A balanced gut microbiota can help maintain an optimal immune response, while dysbiosis can trigger pro-inflammatory cytokines, leading to an increase in osteoclast activity and bone resorption. The inflammatory and anti-inflammatory properties are derived in part from metabolites synthesized in the gut and depend on various factors, including their concentration, the presence of receptors such as toll-like receptors (TLRs), nod-like receptors (NLRs), G protein-coupled receptors (GPCRs) and farnesoid X receptor (FXR), and the interactions with other signaling molecules (93, 94). For example, bile acids can exhibit anti-inflammatory properties by activating the FXR pathway, which in turn suppresses the production of pro-inflammatory cytokines. Such stimulation of the FXR pathway induces the expression of the Runx2 and β catenin genes that promote differentiation of osteoblasts (95). Bile acid induces inhibition of osteoclastogenesis and can be achieved via activation of the FXR pathway (downregulation of c-Fos and NFATc1) or the G-protein coupled bile acid receptor (TGR5) (phosphorylation of AMP-activated kinases) (96, 97).

**Figure 3:**
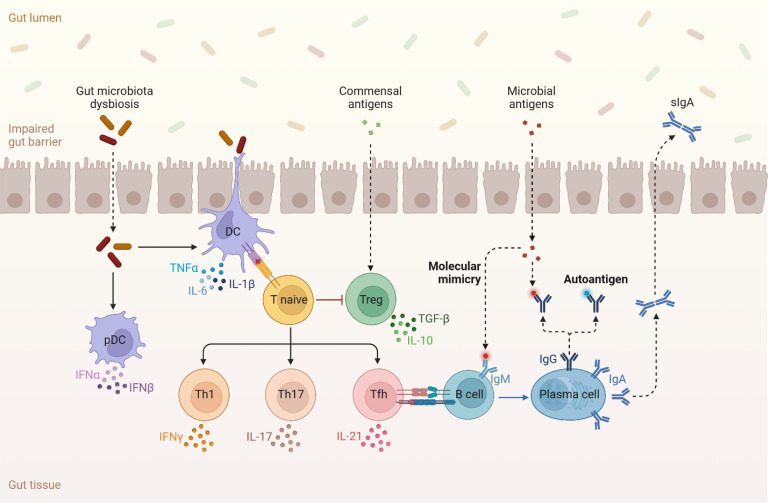
Dysbiosis in the gut microbiome induces the production of multiple pro-inflammatory cytokines such as TNF-α, IL-6, IL-17 amongst others. The availability of these inflammatory molecules and activated T cells inhibit Tregs and their production of cytokines such as TGF-β and IL-10 that reduce bone loss. Lastly, antigens that are produced in the gut microbiome can perturb the normal regulatory function of the immune system and stimulate autoimmune activity.

Bile acids can also bind to the TGR5 receptors on cells belonging to both the innate and adaptive immune system and contribute to immune cell homeostasis by stimulating the production of anti-inflammatory cytokines such as IL-10 and inhibiting the production of inflammatory cytokines such as IL-6 and TNF-α (98, 99) (**Figure 3**). Another way that bile acids can modulate the host immune system is by inducing differentiation of regulator T cells (Tregs) and suppressing the differentiation of Th17 cells (100). Tregs are major inhibitors of bone loss through several mechanisms including the production of IL-4, IL10 and TGF-β1 cytokines and inhibiting differentiation of monocytes into osteoclasts under both *in vitro* and *in vivo* conditions (101) (**Figure 3**). Tregs can also inhibit the effector function of Th17 cells and prevent inflammation-induced bone loss (102–104). This Tregs effect is also induced via suppression of MCSF and RANKL.

Th17 cells are responsible for initiating and stimulating bone resorption by secreting either RANKL or IL-17 (75, 101). IL-17 functions as an osteoclastogenic cytokine that induces the expression of RANKL on osteoclastogenesis supporting cells and osteoblasts (75). Additionally, IL-17 promotes the production of inflammatory cytokines such as TNF-α, IL-1, and IL-6 that all reenforce RANKL production (105). Th17 cells are also capable of promoting bone loss by stimulating osteoclastogenesis (75). Inflammatory cytokines, such as TNF-α, IL-1, and IL-6, can influence bone remodeling by increasing osteoclast activity and bone resorption, leading to reduced bone density. These cytokines can be produced in response to the presence of certain gut microbiome-derived metabolites, as well as other factors like infections or inflammation (75) (**Figure 3**).

## Gut metabolism and bone health

The gut microbiome synthesizes a variety of metabolites and signaling molecules that can influence bone mineralization. The diverse microbial cell population in the gut microbiome contributes to a unique metabolic landscape, with each microbe housing a distinctive set of genes that enable them to catalyze reactions that host cells are unable to perform (55, 71). This metabolic diversity leads to the generation of a wide array of microbiome-derived metabolites. Some of these metabolites, such as short-chain fatty acids (SCFAs), have been shown to play essential roles in maintaining bone health. SCFAs, mainly produced by the fermentation of dietary fibers by gut bacteria, can modulate bone remodeling by influencing the activity of osteoblasts (bone-forming cells) and osteoclasts (bone-resorbing cells) (106). Additionally, SCFAs have anti-inflammatory properties that can help mitigate inflammation-induced bone loss (107). Montalvany-Antonucci et al. showed that the binding of SCFAs to the free fatty acid receptor 2 (FFAR2) can promote bone formation in animals that were fed a high fiber diet, unlike the partial reversal of bone loss observed in FFAR2 -/- mice that were fed a similar high fiber diet. In a more recent study, Wallimann et al. (108) demonstrated the dynamic impact of butyrate on bone integrity. In this study butyrate was observed to significantly reduce osteoclast formation and resorption activity, to lower the abundance of monocytes in bone marrow, and to reduce circulating proinflammatory IL-6 levels in mice.

Other microbiome-derived metabolites, such as bile acids, indoles, and polyamines, also contribute to bone health. Bile acids are generated in the liver and modified by gut bacteria, impacting bone remodeling through interactions with specific receptors on bone cells. For instance, bile acids can bind to the G-protein-coupled bile acid receptor (GPBAR1, also known as TGR5) (109) and the farnesoid X receptor (FXR) on osteoblasts (110) and osteoclasts (111, 112), influencing their differentiation, activity, and overall bone metabolism. Indoles, derived from the metabolism of tryptophan by gut bacteria, have been shown to influence bone remodeling by modulating the differentiation and activity of osteoclasts (113). This is achieved in large part because indole metabolites can exert both pro-inflammatory and anti-inflammatory effects on bone cells. Structurally similar metabolites such as indole-3aldehyde (IAld) and indole-3-acetic acid (I3AA) can initiate divergent bioactivities in bone (113). IAld but not I3AA, was shown to inhibit the expression of pro-inflammatory cytokines (IL-1β, and IL-6), and exhibited pro-osteoclastogenic activity. Collectively, the bioavailability of IAld and I3AA have been implicated in the development and progression of Rheumatoid arthritis (RA).

Polyamines, another class of metabolites are synthesized in the gut through the metabolism of amino acids, are required for various biological processes in the body such as cell growth, proliferation, and survival (114). Polyamines, such as spermine, spermidine, and putrescine, are necessary for proper maintenance of bone health as they can prevent RANKL-induced osteoclastic differentiation in association with the inhibition of nuclear factor-kappa B (NF-κB) in osteoclasts (115, 116). It has been reported that polyamine supplementation inhibits bone loss via the suppression of osteoclast differentiation and proliferation (116). Polyamines also have a prominent role in maintaining gut integrity, which in turn impacts the regulation of bone health. Polyamines regulate the epithelial barrier of the gut by activating the transcription factor c-Myc, which in turn upregulate the expression of tight junction protein E-cadherin (117). Polyamine also regulate the gut barrier by stimulating the expression of TLR2 on intestinal epithelial cells (118).

## Chronic diseases, gut microbiome perturbations, and osteoporosis

Several chronic diseases have been associated with an increased risk of developing osteoporosis, including inflammatory bowel disease (IBD), diabetes, rheumatoid arthritis, and celiac disease (119–121). These conditions negatively impact bone health through mechanisms including chronic inflammation, hormonal imbalances, and nutrient malabsorption (122). Moreover, the presence of these chronic diseases can also lead to perturbations in the gut microbiome, which can further contribute to the development and progression of osteoporosis (123–125). These perturbations can disrupt the delicate balance between bone formation and resorption, ultimately leading to bone loss and increased risk of osteoporosis. The molecular mechanisms underlying this relationship involve the production of microbiome-derived SCFAs, vitamin B12, flavonoids, incretins, serotonin, and IGF-1 (121, 123).

In the context of chronic diseases, gut microbiome perturbations can exacerbate inflammation, leading to increased production of pro-inflammatory IL-1, IL-6, and TNF-α that stimulate osteoclast activity and bone resorption (121). Additionally, chronic diseases can also impair the absorption of essential nutrients, such as calcium and phosphorus, which are critical for bone mineralization.

## Examples of specific chronic diseases and their effects on the gut microbiome and osteoporosis

Chronic diseases such as Inflammatory Bowel Disease (IBD, including Crohn’s disease and ulcerative colitis), rheumatoid arthritis, diabetes (type 1 and type 2), Celiac disease, and others are all characterized by chronic inflammation of the gastrointestinal tract (16, 17). This inflammation can drive gut dysbiosis, nutrient malabsorption, and increase production of pro-inflammatory cytokines, all of which can contribute to bone loss and an increased risk of osteoporosis.

Individuals with type 1 diabetes mellitus (T1DM) have persistently low BMD which is associated with increased risk of bone fractures compared to age, sex, and body mass index (BMI) matched controls (126). The suppressed bone formation and mineralization is thought to be due to hyperglycemia, hypoinsulinemia, autoimmune inflammation, low levels of insulin-like growth factor-1 and vitamin D. In contrast to T1DM, individuals with T2DM have increased BMD but are also at increased risk of bone fractures (127). T2DM induces systemic changes including inflammation, hormonal imbalance, generation of reactive oxygen species, and accumulation of advanced glycation end products which are deleterious to bone health (128).

Genetically susceptible individuals suffering from Celiac disease experience damage to their small intestine that contributes to nutrient malabsorption, including calcium and other essential nutrients required for bone health. They also have significantly lower BMD compared to age, sex, BMI matched T1DM controls (126).

Rheumatoid Arthritis (RA) is an autoimmune disease characterized by chronic inflammation and joint destruction. In addition to the direct effects of inflammation on bone health, RA has also been associated with alterations in the gut microbiome, which can further contribute to bone loss and increased risk of osteoporosis.

## Impact of orally administered drugs on the gut microbiome

Orally administered drugs can have significant effects on the gut microbiome, potentially altering microbial diversity, physiology, and signaling (63). These changes can, in turn, affect bone health and contribute to the development or exacerbation of osteoporosis. Antibiotics are widely used to treat bacterial infections and can have profound effects on the gut microbiome. While they are essential for eliminating pathogenic bacteria, antibiotics can also disrupt the balance of beneficial gut bacteria, leading to a reduction in microbial diversity and compositional changes. This disruption can have negative consequences on bone health, including the alteration of calcium and vitamin D absorption and the production of microbiome-derived metabolites and immune signaling molecules, ultimately contributing to bone loss and osteoporosis. In their work involving the use of narrow-spectrum antibiotics Luna et al. were able to demonstrate that in male C57BL/6J mice continuous exposure to neomycin whole bone strength was reduced even though no observable differences were detected in histological and serum markers of bone turnover in control and treated animals (129). Similarly, Rios-Arce et al. demonstrated that two weeks of treatment with neomycin and ampicillin antibiotics induces bone loss in multiple strains of mice (89). In addition to the aforementioned antibiotics, antifungal agents such as fluconazole can also impact the gut microbiome by reducing the diversity of fungal species within the gastrointestinal tract (130, 131). Although the effects of antifungal agents on bone health are less well-studied than antibiotics, it is possible that they could contribute to osteoporosis through similar mechanisms, such as the alteration of microbiome-derived metabolites and immune signaling molecules.

Non-antimicrobial drugs drive dysbiosis in the gut microbiome and trigger osteoporosis. Proton pump inhibitors (PPIs) are widely prescribed for the treatment of acid-related gastrointestinal disorders, such as gastroesophageal reflux disease (GERD) and peptic ulcers (132). PPIs can alter the gut microbiome by reducing stomach acid levels, which can lead to an overgrowth of certain bacterial species in the upper gastrointestinal tract. These changes in microbial composition can impact bone health by affecting the absorption of calcium, iron, magnesium, folate, biotin, vitamin B12, and other essential nutrients, as well as the production of microbiome-derived metabolites and immune signaling molecules (132–134).

Nonsteroidal anti-inflammatory drugs (NSAIDs) NSAIDs are commonly used to treat pain and inflammation, but their long-term use has been associated with an increased risk of osteoporosis. NSAIDs can alter the gut microbiome by disrupting the balance of beneficial bacteria and promoting the growth of potentially harmful species (135–137). These changes in microbial composition can lead to an increase in gut permeability, allowing microbiome-derived metabolites and immune signaling molecules to enter systemic circulation, which can negatively affect bone health (138, 139).

Selective serotonin reuptake inhibitors (SSRIs) are widely prescribed for the treatment of depression and anxiety disorders. Emerging evidence suggests that SSRIs can also impact the gut microbiome by altering the composition and diversity of microbial communities (140, 141). While the exact mechanisms linking SSRIs, gut microbiome, and bone health are not yet fully understood, it is possible that these drugs could contribute to osteoporosis through the dysregulation of microbiome-derived metabolites and immune signaling molecules. Gut derived serotonin is known to reduce osteoblast proliferation and drive bone loss (142, 143). Thus, as SSRI use induces disruptions in the composition and diversity of the gut microbiota, the bioavailability of brain derived and gut derived serotonin will be skewed, leading to alterations in osteogenic and osteoclastic activity. Antidepressants such as fluoxetine are capable of disrupting sphingolipid metabolism in bone marrow tissue which has been shown to contribute to increases in the secretion of RANKL, a key inducer of osteoclastogenesis and bone loss (144). Newer antidepressants such as arketamine can ameliorate reductions in bone mineral density in animal models (145).

Glucocorticoids are a class of corticosteroid hormones commonly prescribed for the treatment of inflammatory and autoimmune diseases, such as rheumatoid arthritis and asthma (146). Long-term use of glucocorticoids is known to increase the risk of osteoporosis by directly affecting bone metabolism (147–149). However, emerging evidence also suggests that glucocorticoids can impact the gut microbiome by altering microbial composition, diversity, and metabolism in several animal models (150–152). These changes in the gut microbiome may exacerbate the negative effects of glucocorticoids on bone health, contributing to the development or progression of osteoporosis.

In addition to the inflammatory mechanisms compromising bone health in individuals with diabetes, certain medications used to treat diabetes such as thiazolidinediones can also adversely affect bone health. Thiazolidinediones induce preferential differentiation of mesenchymal stem cells into adipocytes rather than osteoblasts, and promote osteoclast differentiation; thus impairing bone formation (153, 154). Another antidiabetic drug, metformin, is capable of regulating bone remodeling via its inhibitory effect on osteoclast activation (155). Metformin has been shown to impact on osteogenic induction of bone marrow progenitor cells and bone repair in animal models (156, 157). In rat models of type 2 diabetes with induced parietal bone lesions oral metformin was shown to increase bone lesion repair and reossification in both aged and streptozotocin-induced diabetic rats compared to controls (157).

## Intervention strategies and future directions

The growing understanding of the complex interplay between the gut microbiome, orally administered drugs, and bone health presents an opportunity for the development of targeted therapeutic strategies for the prevention and treatment of osteoporosis. By modulating the gut microbiome, it may be possible to improve bone health and mitigate the negative effects of certain medications on osteoporosis risk.

Probiotics are live microorganisms that, when administered can confer health benefits to the host. Several studies have suggested that probiotics, particularly those containing *Lactobacillus* and *Bifidobacterium* strains, can improve bone health by modulating the gut microbiome, enhancing nutrient absorption, and reducing inflammation (158–160). Probiotic supplementation may be a promising strategy for the prevention and treatment of osteoporosis, particularly in individuals with altered gut microbiome due to chronic diseases or medication use. The consumption of probiotics helps to limit dysbiosis in the gut microbiome via regulation of the ratio of *Firmicutes/Bacteroides* and other bacterial species, as well as maintenance of the Treg-Th17 cell balance that is needed to prevent bone loss (161–164).

Prebiotics are non-digestible food components, such as dietary fiber and oligosaccharides, that selectively stimulate the growth and activity of beneficial gut bacteria (165). Prebiotic supplementation can help restore and maintain a healthy gut microbiome and promote the production of microbiome-derived metabolites, such as short-chain fatty acids that are key regulators bone health (166). Incorporating prebiotics into the diet may be an effective strategy for preventing and treating osteoporosis, especially in populations at risk due to gut microbiome perturbations (167, 168). Prebiotics can increase bone mineral density by decreasing intestinal permeability and systemic inflammation, and by increasing the expression of Ca transporters in the intestine (169). Fecal microbiota transplantation (FMT) involves the transfer of fecal material from a healthy donor to a recipient with an altered gut microbiome, with the goal of restoring a healthy microbial community. Although FMT has been primarily used for the treatment of recurrent *Clostridium difficile* infection, there is growing interest in its potential for treating other conditions associated with gut microbiome dysbiosis, including osteoporosis (75). Significant increases in the markers of bone health (volume fraction, trabecular number, thickness) have been observed in senile rat models treated via FMT (90). Further research is needed to explore the efficacy and safety of FMT for osteoporosis treatment, as well as the optimal donor selection and administration protocols.

Personalized nutrition and lifestyle interventions tailored to an individual’s unique gut microbiome may be an effective approach for preventing and treating osteoporosis. By assessing an individual’s gut microbial composition and functional capacity, personalized dietary and lifestyle recommendations can be developed to promote a healthy gut microbiome and optimize bone health. Such interventions may include the consumption of specific prebiotic-rich foods, the incorporation of probiotic supplements, and the adjustment of medications that adversely affect the gut microbiome and bone health.

## Conclusion

The field of osteomicrobiology has made significant progress in recent years, shedding light on the complex interactions between the gut microbiome, orally administered drugs, and osteoporosis. However, there are still many challenges and unanswered questions that need to be addressed in order to fully understand these interactions and develop effective therapeutic strategies for osteoporosis prevention and treatment.

While it is clear that many orally administered drugs can impact the gut microbiome, the exact mechanisms by which these medications alter microbial composition, diversity, and function has been elucidated for a fraction of FDA approved therapeutics. Additionally, the bidirectional relationship between medications and the gut microbiome, wherein the microbiome can also affect drug metabolism and efficacy, adds another layer of complexity to these interactions. Future research should aim to unravel these complex interactions to inform the development of targeted drug therapies that minimize negative effects on bone health.

Although various therapeutic strategies targeting the gut microbiome, such as probiotics, prebiotics, and fecal microbiota transplantation, have shown promise for the prevention and treatment of osteoporosis, many challenges remain. Further research is needed to optimize these approaches, determine their long-term safety and efficacy, and identify the most effective combinations of interventions for specific patient populations.

## Author contributions

SB and KF acquired and analyzed available data and contributed to drafting and writing of this manuscript. All authors approved the submission of this version. All authors contributed to the article.
